# The Effect of Sepsis on the Erythrocyte

**DOI:** 10.3390/ijms18091932

**Published:** 2017-09-08

**Authors:** Ryon M. Bateman, Michael D. Sharpe, Mervyn Singer, Christopher G. Ellis

**Affiliations:** 1 Department of Medical Biophysics, University of Western Ontario, London, ON N6A 5C1, Canada; cgellis@uwo.ca; 2 Department of Anesthesia and Critical Care Western, University of Western Ontario, London, ON N6A 5C1, Canada; Michael.Sharpe@lhsc.on.ca; 3 Research Department of Clinical Physiology, Division of Medicine, University College London, London WC1E 6BT, UK; m.singer@ucl.ac.uk; 4 Bloomsbury Institute of Intensive Care Medicine, University College London, London WC1E 6BT, UK

**Keywords:** sepsis, erythrocyte, rheology, morphology and microcirculation

## Abstract

Sepsis induces a wide range of effects on the red blood cell (RBC). Some of the effects including altered metabolism and decreased 2,3-bisphosphoglycerate are preventable with appropriate treatment, whereas others, including decreased erythrocyte deformability and redistribution of membrane phospholipids, appear to be permanent, and factors in RBC clearance. Here, we review the effects of sepsis on the erythrocyte, including changes in RBC volume, metabolism and hemoglobin’s affinity for oxygen, morphology, RBC deformability (an early indicator of sepsis), antioxidant status, intracellular Ca^2+^ homeostasis, membrane proteins, membrane phospholipid redistribution, clearance and RBC O_2_-dependent adenosine triphosphate efflux (an RBC hypoxia signaling mechanism involved in microvascular autoregulation). We also consider the causes of these effects by host mediated oxidant stress and bacterial virulence factors. Additionally, we consider the altered erythrocyte microenvironment due to sepsis induced microvascular dysregulation and speculate on the possible effects of RBC autoxidation. In future, a better understanding of the mechanisms involved in sepsis induced erythrocyte pathophysiology and clearance may guide improved sepsis treatments. Evidence that small molecule antioxidants protect the erythrocyte from loss of deformability, and more importantly improve septic patient outcome suggest further research in this area is warranted. While not generally considered a critical factor in sepsis, erythrocytes (and especially a smaller subpopulation) appear to be highly susceptible to sepsis induced injury, provide an early warning signal of sepsis and are a factor in the microvascular dysfunction that has been associated with organ dysfunction.

## 1. Introduction

Sepsis has recently been defined as “a life-threatening organ dysfunction caused by a dysregulated host response to infection” [1]. This dysregulation is particularly evident in the cells, organelles and overall system involved in the delivery and consumption of oxygen, or more specifically, the erythrocytes [2,3,4,5,6,7,8], mitochondria [9,10,11,12] and microcirculation [13,14,15,16]. In this review, we consider the effect of sepsis on the red blood cell (RBC). We place the RBC in the context of a dysfunctional microcirculation and consider RBC exposure to exogenous and endogenous reactive oxygen species, used by the body to fight off infection or generated by hemoglobin, and maximized under hypoxic conditions. And, we discuss the effect of sepsis on the RBC with respect to distribution width, hemoglobin oxygen binding, morphology, rheology, the sources of reactive oxygen species that impinge on the RBC and their effects, alterations in intracellular Ca^2+^ homeostasis, the effect of virulence factors, alterations in membrane proteins and impaired erythrocyte O_2_-dependent adenosine triphosphate (ATP) efflux under hypoxic conditions. PUBMED was used to source the primary articles cited in the review and Google Scholar Citations was used to cross-reference and source secondary articles found by reviewing the articles citing the primary articles. There were no size restrictions on the clinical studies cited.

**Figure 1:**
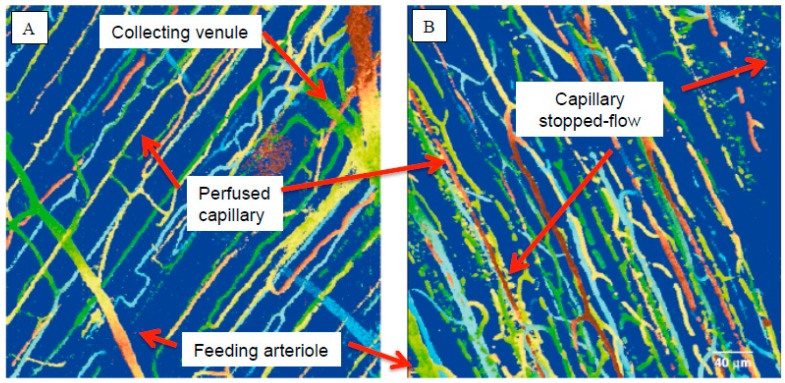

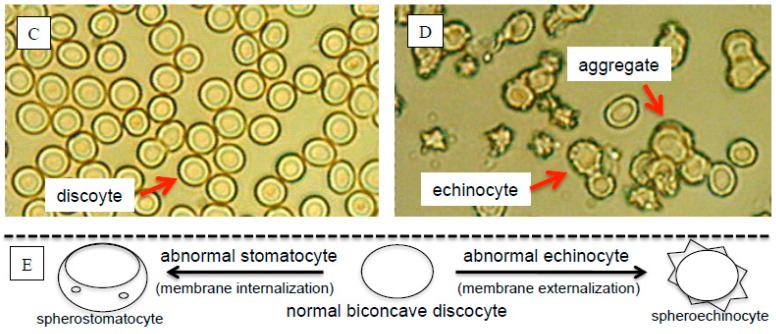
Sepsis alters erythrocyte morphology and blood flow in capillary networks. (**A**,**B**) Intravital multiphoton images of the extensor digitorum longus (EDL) hindlimb skeletal muscle microcirculation of a control and septic mouse, 5 h after the onset of septic injury, respectively (Bateman et al. [34]). Solid lines indicate perfused capillaries, whereas broken lines indicate stopped-flow capillaries. Capillaries were pseudo colored for depth below the surface: brown (surface), green (75 µm), blue (150 µm) (Bateman et al. [35]). (**C**,**D**) Blood smears from a healthy volunteer with normal red blood cell (RBC) discocyte morphology and a septic shock patient with abnormal RBC morphology, respectively. Note the presence of echinocytes (also reported by de Oliveira et al. [4]), RBC aggregates and abnormal RBCs in the septic patient [8] (Copyright from John Wiley and Sons and Copyright Clearance Center). (**E**) The range of abnormal RBC morphologies, from spherostomatocyte to spheroechinocyte, described by Reinhart et al. [26], where damaged RBC membranes can either be internalized forming vacuoles or externalized forming spikes, respectively, as seen in the blood smear of septic RBCs.

Figure 1A,B shows images of the effect of sepsis on the mouse hindlimb skeletal muscle microcirculation during the onset of sepsis. What is immediately apparent from the images is that sepsis induced microvascular dysregulation is: (1) an early onset phenomenon (changes in capillary blood flow distribution are clearly evident by 5 h); and (2) that it is not uniform throughout the tissue (as some tissue regions suffer from a complete loss of capillary flow, whereas other regions maintain some capillary flow). Accordingly, RBCs are exposed to different microenvironments depending on their location at any particular time. This heterogeneity, or maldistribution [14,15] of RBC capillary flow is a hallmark of sepsis, both experimentally and clinically, and has been observed in a variety of septic microvascular beds including, but not limited to, the skeletal muscle [13,14,15,17,18,19], diaphragm [20], myocardium [21] and sublingual microcirculations [16,22]. Mathematical models [23] of local tissue oxygen levels based on measurements of capillary RBC hemoglobin O_2_ saturation (capillary RBC SO_2_ [14]) indicate that sepsis exposes both the local tissue and the trapped RBCs to significant regions of hypoxia. This local hypoxia is significant because it can increase RBC oxidant stress [24] and decrease RBC antioxidant capacity [25].

Figure 1C,D shows images of blood smears taken from an anemic septic patient [8]. What is immediately apparent from the slides is that sepsis can have profound effects on both RBC morphology and RBC aggregation. The presence of echinocytes (RBCs with multiple spikes protruding from their surface, reported in this and other studies [4]), disintegrating RBCs and erythrocyte aggregates (or clumps) shows that sepsis dramatically alters the cytoskeleton, membrane and membrane surface properties of the RBC. Figure 1E depicts the spectrum of abnormal RBC morphologies observed by Reinhart and Chien [26]. The authors reported that the erythrocyte could be induced to either internalize or externalize its membrane, resulting in abnormal stomatocyte–echinocyte transformations. More recent clinical studies have revealed that sepsis can induce both stomatocyte and echinocyte formation in septic patients [4,7,8].

Not apparent from the slides, however, is that sepsis also alters the affinity of hemoglobin for the oxygen molecule, as well as, the mechanical properties of the RBC (manifested by a decrease in RBC deformability). This hardening of the RBC is associated in animal models with changes in microvascular capillary flow and loss of functional capillary density in septic skeletal muscle [3], RBC entrapment [27] and clearance [28] in control and infected animals, respectively. Clinically and experimentally, a change in RBC deformability is an early indicator of sepsis [3,29,30,31,32,33].

## 2. Erythrocyte Size, Shape and Deformability

Under normal physiological conditions, the mature erythrocyte has a characteristic biconcave disc shape, approximately 6–8 µm in diameter and 2 µm thick [36]. Using a micropipette technique, Linderkamp et al. [37] found that the RBC surface area was 39% in excess of what was needed to enclose its cellular volume. This extra surface area allows the RBC to adopt various shapes at constant volume [36], as it tumbles and flows through the larger blood vessels and then deforms and bends as it flows through capillary networks with capillary diameters of 5–6 µm or squeezes through the narrow interendothelial slits in the spleen that function to screen RBCs on the basis of their geometry and deformability [38,39]. Subjected to constantly changing oxygenation and shear conditions, the average life span of an RBC is approximately 120 days, where its size (surface area and volume), hemoglobin content and mechanical properties are age related [40,41,42]. Moreover, RBC susceptibility to cell death is also age related [43].

### 2.1. The Effect of Sepsis on Red Blood Cell (Shape) and Distribution Width (RDW)

In a septic patient population characterized by decreased hematocrit (HCT), hemoglobin (Hb) and RBC count, but with no change in RBC mean corpuscular volume (MCV) within the first 24 h of sepsis, Piagnerelli et al. [5] reported RBCs were more spherical in septic patients compared to healthy volunteers. Sphericity was based on forward light scatter and was found to correlate with RBC sialic acid membrane content, suggesting that altered RBC surface charge was a factor in increased RBC sphericity. 

The erythrocyte distribution width (RDW) parameter (coefficient of variation of RBC volume) quantifies the heterogeneity of RBC volume (anisocytosis) as the ratio of the standard deviation of RBC volume to the mean corpuscular volume. It is calculated as part of a routine automated complete blood count (CBC) and has been associated with a number of disease states including cardiovascular disease, cancer and diabetes [44].

Retrospective and prospective studies of critically ill patients have reported that increased RDW is also a strong and independent risk factor of sepsis and septic shock mortality [45,46]. Retrospectively, Sadaka et al. [45] divided septic patients into five groups based on their RDW on Day 1 and found the risk of mortality increased 35.6 and 43.1 fold in patients with elevated RDW values in the 4th and 5th quintiles, respectively. In a similar retrospective study investigating RDW in septic patients with a Gram-negative bacteremia, Ku et al. [47] reported that RDW at the onset of bacteremia was an independent predictor of 28-day mortality. Jo et al. [48] reported that RDW was significantly higher in sepsis and septic shock non-survivors compared to survivors. In a prospective study where sepsis and septic shock patients were stratified by baseline RDW (at time of admission to the emergency department) and changes in RDW between baseline and 72 h, Kim et al. [46] reported that increased RDW from baseline was associated with increased 28 and 90 days mortality in septic shock patients. However, the pathophysiological mechanisms affecting RDW are unknown and there is some uncertainty regarding the significance of the RDW parameter as some studies failed to detect a difference in RDW between septic patients and control group [5,49].

While the mechanism(s) responsible for increased RDW in septic patients have not been elaborated, a number of causative factors have been suggested including inflammation and oxidative stress [50], reduced RBC survival [28,51], altered RBC membrane function [52] and inhibited RBC maturation and release of nucleated RBCs into the circulation [53]. In a non-sepsis age study, RDW was found to negatively correlate with decreased RBC deformability [54], suggesting that increased RDW reflected altered rheology in older subjects. However, Todd et al., have reported that sepsis induced changes in the RBC deformability are unrelated to changes in RBC surface area to volume ratio [55]. Furthermore, in a clinical study of septic shock patients, Fontana et al. [49] reported that they could not detect any relationship between RDW and alterations in sublingual microvascular parameters; moreover, the authors did not find any relationship between RDW and disease severity, septic shock or mortality. Taken together, the conflicting results suggest that more research is required to determine the cause and effect relationships between sepsis-induced alterations in RBC volume heterogeneity and its relationship to sepsis pathophysiology and patient outcome.

### 2.2. Sepsis Induced Changes in RBC Deformability Are Clinically Important

Changes in the mechanical properties of erythrocytes during sepsis, which are manifested by increased RBC rigidity and measured by decreased RBC deformability, are clinically relevant for a number of reasons. First, decreased erythrocyte deformability develops early in rat (3–6 h) and pig (6–8 h) models of sepsis [3,56,57] and has been detected within 24 h of admission of critically ill patients [30,32]. Second, it may provide a means to differentiate between trauma and septic patients [30], as RBC deformability recovered in the former but not in the latter group of patients. Within 24 h of ICU admission, Langenfeld et al. [30] monitored critically ill patients for changes in RBC deformability (RBCD) using a filtration method and detected progressive decreases in RBCD over the next 2–8 days, which preceded infection and appeared to both predict and differentiate trauma patients from sepsis patients. Third, decreased erythrocyte deformability in sepsis is associated with organ dysfunction and patient outcome [29,58,59]. Taken together, these experimental and clinical findings suggest a significant role for RBC injury in sepsis induced microvascular dysregulation and patient outcome.

### 2.3. Septic RBC Subpopulations with Altered Morphology and Decreased Deformability

Several reports have identified subsets of septic RBCs with significantly decreased erythrocyte deformability [2,3,40]. In an 18 h rat cecal ligation and perforation (CLP) model of sepsis, Baskurt et al. [2] interpreted differences in trends between mean and median RBC transit times, measured using a cell transit analyzer (CTA, consisting of thirty 5 μm diameter × 15 µm cylindrical pores) to mean that a small population of RBCs with “extremely long” transit times existed in the septic blood. Such a subpopulation was confirmed in subsequent studies. Using a micropipette aspiration technique to measure RBC membrane deformability at 37 °C in a 6 h rat sepsis model (cecal ligation and perforation, CLP), Bateman et al. [3] detected a small subpopulation of RBCs, (approximately 5% of the total) with reductions in deformability of >50%. In a 24 h CLP mouse model of sepsis, where RBCs were fractionated (elutriation) into subpopulations on the basis of cell size and presumed age, Condon et al. [40] used ektacytometry and the shear stress of the half max deformability index (elongation index) to identify subpopulations of RBCs (approximately 20% of the total population) with significantly reduced RBCD compared to a sham group. Non-sepsis studies have suggested that a subpopulation of RBCs is more susceptible to oxidation resulting in membrane alterations and in vivo destruction [60]. While hallmarks of sepsis are increased capillary stopped flow and decreased erythrocyte deformability, the deformability characteristics of arrested RBCs are unknown.

## 3. Hemoglobin Oxygen Binding and 2,3-Bisphosphoglycerate (2,3-BPG)

The erythrocyte is the fundamental cell involved in the delivery of oxygen from the lungs to the tissues. It carries oxygen bound to hemoglobin. Adult or mature hemoglobin is a tetrameric metalloprotein with a regulatory central cavity. Each hemoglobin subunit contains a porphyrin ring with a central iron atom (the heme-iron cofactor) that can bind an oxygen molecule. Oxygen binds to hemoglobin in a cooperative manner such that it gives rise to the familiar sigmoidal oxygen dissociation curve (ODC, the graphical relationship between hemoglobin oxygen saturation and partial pressure of oxygen, where P_50_ is the partial pressure of oxygen (PO_2_), under standard or in vivo conditions, at which Hb O_2_ saturation is 50%). 

Under normal physiological conditions and low pH, the increased acidity decreases hemoglobin’s affinity for oxygen (Bohr effect), shifting the ODC to the right, increasing the P_50_ and promoting the release of oxygen. This is advantageous in actively respiring tissues, as the carbon dioxide (CO_2_) produced can diffuse into the red cell where it can either bind to hemoglobin or react with water and be interconverted by carbonic anhydrase to hydrogen ion (H^+^) and bicarbonate (HCO^3−^). In sepsis, however, evidence suggests that the RBC ODC is shifted to the left [61,62,63,64], decreasing the P_50_ and increasing hemoglobin’s affinity for oxygen; however, the relationship in sepsis is complex and not completely understood.

### The Effect of Sepsis on RBC 2,3-BPG and the Correlation with Acidemia

Essential to RBC function is a side-branch of glycolysis (Rapoport-Lubering shuttle) that produces 2,3-bisphosphoglycerate. 2,3-BPG (also referred to as 2,3-DPG) is an allosteric regulator of hemoglobin that binds to the central cavity of the hemoglobin molecule and decreases the affinity of hemoglobin for oxygen by a factor of 26. It is present at high concentrations under normal physiology and is synthesized by the RBC under hypoxic conditions [65,66], thereby shifting the ODC to the right, increasing the P_50_ and promoting the release of oxygen. 

There remains some uncertainty, however, as to the effect of sepsis on RBC 2,3-BPG levels [67]. An experimental pilot study using endotoxemic mice suggested that, in the early hypoglycemic phase of endotoxemia, the RBC increased its 2,3-BPG level [68], suggesting the RBC was attempting to release more oxygen in hypoxic regions. However, in critically ill and septic patients, acidemia, hypophosphatemia and transfusion of 2,3-BPG depleted blood are all factors that can shift the ODC to the left, decrease the P_50_ [62], increase Hb affinity for oxygen and decrease oxygen release. Numerous experimental and clinical studies have reported decreased levels of RBC 2,3-BPG and reduced P_50_ including in endotoxemic baboons [68], and critically ill and sepsis patients [61,67,69], respectively.

The primary mechanism in critically ill patients appears to be associated with acidemia [70], and several studies have reported that RBC 2,3-BPG (or P_50_) is correlated with pH [64,65,69,71]. This was supported by Chillar et al. [71] who reported that 2,3-DPG correlated with arterial pH over a range of 6.9 to 7.6 in septic shock patients. Acidemia reduces the affinity of hemoglobin for oxygen (Bohr effect), but over time, also inhibits the synthesis of 2,3-BPG, which opposes the Bohr effect. However, while studies of critically ill and septic patients indicate that 2,3-BPG may decrease [61,69], it may also remain unchanged [67]. Additionally, Ibrahim et al. [67] reported there was no difference in 2,3-BPG levels between ICU survivors and non-survivors. There was, however, a greater variation in 2,3-BPG levels in critically ill patients and RBC 2,3-BPG was found to correlate with plasma acidosis. 

In sepsis, where the microcirculation becomes dysregulated and oxygen diffusion distances increase due to loss of functional capillary density [14,15,21], shifting the ODC to the right would enhance the release of oxygen in hypoxic tissues, whereas shifting the ODC to the left would seemingly be detrimental, as RBCs would retain more oxygen in hypoxic regions. However, further research is required to gain a more complete understanding of sepsis induced shifts in the RBC ODC and the effect on oxygen release from capillary networks in hypoxic tissue [13,14,23,72].

## 4. Sepsis Induced Oxidative Stress, Effects on Erythrocytes and the Importance of Antioxidants

Sepsis has profound effects on all aspects of oxygen physiology, from its transport in RBCs and its distribution via the microcirculation to its ultimate utilization in the mitochondria to produce energy in the form of ATP. In addition, of seemingly critical importance to the septic patient is the conversion of oxygen into oxygen free radicals, reactive oxygen species and oxidizing species, as Marik et al. [73] reported that treating septic patients with vitamin C (a small molecule antioxidant), thiamine and hydrocortisone prevented organ dysfunction and decreased mortality. While the mechanism is uncertain, vitamin C has been reported to inhibit RBC cell destruction via eryptosis [74]. Similarly, vitamin E (a small molecule antioxidant) was found to improve septic patient outcome [75] and improve RBC deformability. 

### 4.1. Sepsis Induced Reduced Antioxidant Status

Antioxidants protect cells from the damaging effects of oxygen free radicals and reactive oxygen species (superoxide anion, hydrogen peroxide, hyroxyl radical) which cause oxidative damage to membranes (lipid peroxidation), proteins (denature and oxidize amino acid residues and thiol groups) and DNA (introduce strand breaks) resulting in cell and organelle damage and altered cell function, especially, if endogenous antioxidant defenses are depleted due to infection or sepsis [73,76,77,78,79,80,81,82]. 

In support of the concept of sepsis induced *reduced antioxidant status*, Goode et al. [76] reported that septic shock patients had decreased plasma levels of retinol (vitamin A), α-tocopherol (vitamin E), β-carotene and lycopene (indicative of decreased small molecule antioxidant potential), and those patients suffering from organ failure had increased evidence of lipid peroxidation. Consistent with a mechanism whereby vitamin E prevents lipid peroxidation, Richard et al. [81] reported that lipid peroxidation was inversely related to plasma vitamin E levels in patients with adult respiratory distress syndrome (ARDS). In agreement, the plasma profile from septic patients with acute renal failure [83] included increased malonyldialdehyde (MDA) (indicative of increased lipid peroxidation), decreased ascorbic acid and α-tocopherol (indicative of decreased small molecule antioxidant potential), increased plasma catalase (indicative of increased plasma conversion of superoxide anion to hydrogen peroxide), and decreased RBC glutathione (indicative of reduced potential to recycle oxidized antioxidants and protect protein thiol groups from being oxidized). 

Similarly, in patients with acute respiratory distress syndrome monitored over six days, Metnitz et al. [79] reported that the antioxidant system was “severely compromised”. In plasma, they found elevated levels of MDA and decreased levels of ascorbate, α-tocopherol, β-carotene and selenium. However, while neutrophil production of superoxide anion and hydrogen peroxide was found to decrease over time, no changes in the RBC antioxidants which detoxify superoxide anion and hydrogen peroxide (superoxide dismutase (SOD), catalase (CAT) or glutathione peroxidase (GPx)) were detected, respectively. Nevertheless, the potential significance of low levels of the small molecule antioxidants, vitamin E and vitamin C, and their repletion, on the erythrocyte in terms of altered RBC deformability and patient survival is suggested from studies by Powell et al. [75] and Marik et al. [73], which reported improved RBC deformability and survival with vitamin E treatment and prevention of progressive organ dysfunction with vitamin C treatment, respectively.

In support of sepsis induced reduced antioxidant status, an earlier study using the total peroxyl radical trapping (TRAP) method [84], which measured the reaction of peroxyl radicals with antioxidants as a means of assessing antioxidant capacity, reported that antioxidant capacity was decreased in sepsis patients within 24 h of diagnosis. Moreover, and somewhat paradoxically, the authors found that the TRAP signal increased in septic shock non-survivors over an 8-day period. However, the increased TRAP signal was largely due to an increase in bilirubin, the end product of heme-degradation. Accordingly, and of potential significance in these septic shock patients is that hemoglobin oxidation may have initiated heme-degradation, suggesting that increased antioxidant status in the septic shock non-survivors may have come at the expense of RBC viability. 

In a related study by Doise et al. [82], where total plasma antioxidant capacity (TAC) was measured they found no difference in TAC levels at the outset (patients recruited within 18 h of onset of septic symptoms) between healthy subjects and those with severe sepsis or septic shock. However, the small molecule antioxidants α-tocopherol (vitamin E/lipid ratio), retinol and ascorbate (consistent with hypovitaminosis C in patients with an acute phase response [77]) were significantly decreased (and highly depleted in the case of vitamin C) in both sepsis groups compared to control. 

### 4.2. Activated Neutrophils and Endothelial Cells as Sources of Reactive Oxygne Species (ROS)

In isolated neutrophils, Dewas et al. [85] reported that sepsis-induced pro-inflammatory cytokines (TNF-α, IL-1 and G-CSF) activated the assembly of reduced nicotinamide adenine dinucleotide phosphate (NADPH) oxidase on the outer cell membrane and triggered the subsequent “respiratory burst” [86], generating powerful reactive oxygen species (ROS) that are used to destroy engulfed bacteria. Similarly, sepsis upregulates NADPH oxidase in vascular endothelial cells, where they can release oxygen free radicals into the vascular space [18] and impinge erythrocytes. NADPH oxidase generates the oxygen free radical superoxide anion (O_2_^−^) from oxygen and NADPH. Superoxide is then converted to hydrogen peroxide (H_2_O_2_) by superoxide dismutase (SOD) and used in combination with other oxidants to kill bacteria, but which can escape the neutrophil and damage adjacent tissue and RBCs [87]. 

This was demonstrated in two related in vitro studies where human whole blood was challenged with *Escherichia coli* (*E. coli*) endotoxin. Todd et al. [88,89] reported that endotoxin increased RBC intracellular Ca^2+^ (discussed in greater detail in Section 5) and membrane viscosity, while decreasing RBC deformability (a marker of sepsis as discussed earlier). Furthermore, removal of leukocytes or treatment with oxygen free radical scavengers attenuated the deleterious effects of *E. coli* endotoxin on the RBC, linking RBC exposure to leukocyte ROS with RBC pathophysiology. 

Additionally, Weiss [90,91] found activated human neutrophils were capable of releasing superoxide and damaging RBCs and that activated neutrophils in contact with RBCs, but independent of phagocytosis, were able to generate reactive oxygen species that were capable of oxidizing hemoglobin to methemoglobin and other hemoglobin oxidation products. Inhibition studies suggested the superoxide anion was entering the RBC via the Band 3 anion channel, whereas hydrogen peroxide was apparently diffusing across the RBC membrane. A combination of antioxidant enzymes, superoxide dismutase (scavenges superoxide anion) and catalase (scavenges hydrogen peroxide) protected the RBCs from oxidative damage. More recently, de Oliveira et al. [4] showed in a series of in vitro experiments that neutrophils in contact with oxidatively damaged human RBCs resulted in increased reactive oxygen species production that could be inhibited by superoxide dismutase.

### 4.3. The Erythrocyte as a Source of Reactive Oxygen Species

Erythrocytes themselves can also be a source of reactive oxygen species. In vitro studies have demonstrated that hemoglobin autoxidation results in the production of superoxide anion, which can be readily converted by superoxide dismutase to hydrogen peroxide and escape the RBC. The hemoglobin autoxidation reaction [24,92] is maximized at hemoglobin oxygen saturations of approximately 60% (RBC SO_2_) [24]. The potential significance in sepsis is that these reduced hemoglobin oxygen saturations conditions are known to exist in RBCs at the venular-ends of capillaries in the hindlimb skeletal muscle of control and septic rats [13,14,23]). The significance in sepsis is that trapped RBCs in stopped flow capillaries (see Figure 1) may become local sources of tissue oxidant stress as RBCs desaturate in these hypoxic microenvironments and generate reactive oxygen species. However, more research is required to test the hypothesis and establish the in vivo effects of RBC generated ROS.

### 4.4. Plasma Xanthine Oxidase as a Source of Reactive Oxygen Species

Another source of oxidative stress for the RBC is plasma xanthine oxidase, an enzyme that generates superoxide anion. In one study, sepsis non-survivors were found to have elevated plasma levels of xanthine oxidase (XO) activity and protein carbonyl formation (marker of protein oxidation) compared to healthy volunteers [93]. However, by 72 h there was no difference in plasma XO activity between survivors and non-survivors; though, evidence of plasma lipid peroxidation and oxidative damage was greater in septic non-survivors, suggesting the non-survivors had experienced greater levels of oxidant stress, as would have had their circulating erythrocytes, or had lower levels of antioxidant defenses. 

### 4.5. Effect of Oxygen Free Radicals and Reactive Oxygen Species on the Erythrocte

While definitive studies on the in vivo effects of specific sepsis-induced reactive oxygen species (superoxide, hydrogen peroxide and hydroxyl radical) on erythrocyte properties are lacking, in vitro experiments provide some insight into possible mechanisms. In vitro experiments have found that oxygen free radicals alter proteolytic susceptibility and RBC rheology, by degrading the integral membrane protein band 3 (anion channel) and the cytoskeleton protein, spectrin, while decreasing RBC deformability [94]. An intriguing study using isolated RBCs by Baskurt et al. [95] investigating the effects of endogenous and exogenous superoxide anion on RBC rheology found that RBCs exposed to extracellular superoxide anion had increased RBC aggregation, whereas intracellular production of superoxide anion resulted in decreased RBC deformability.

In vitro experiments have also shown that incubating human RBCs with hydrogen peroxide induces numerous deleterious effects on the RBC (in a dose-dependent manner), including spectrin-hemoglobin cross-linking, echinocyte formation (see Figure 1), decreased membrane phosphatidylcholine access, increased RBC adhesion, increased monocyte phagocytosis, and decreased RBC deformability [96]. Similarly, Srour et al. [97] found that human RBCs exposed to H_2_O_2_ had altered morphology (echinocyte shape change) and rheology (decreased RBC defomability) that was proportional to both MDA (lipid peroxidation) and protein degradation. RBCs were also more osmotically fragile. 

In a follow-up study, McKenney et al. [60] transfused hydrogen peroxide treated RBCs into a Baboon model and found there was a decrease in 24 h RBC survival time. The clearance of oxidatively damaged RBCs correlated with both spectrin-hemoglobin complex formation and decreased RBC deformability. Additionally, a subpopulation of dense, dehydrated RBCs was found to be more susceptible to oxidant damage. In support of this finding, an earlier study by Jain et al. [98] found that rabbit RBCs exposed to MDA (MDA is a dialdehyde cross-linking species produced by lipid peroxidation and elevated levels are associated with sepsis) had decreased RBC deformability that was negatively correlated with both MDA and phospholipid adducts. At low MDA levels, only RBC deformability was affected, and, when these RBCs were infused into the donor rabbit, they were rapidly cleared from the circulation. At higher MDA levels, exposed RBCs showed K^+^ loss, consistent with potassium leak [99], cell dehydration and increased intracellular viscosity.

### 4.6. Effect of Sepsis on Erythrocyte Antioxidants

Given that RBCs are continuously exposed to endogenous hydrogen peroxide (H_2_O_2_) under normal conditions (autoxidation) and acutely exposed to exogenous H_2_O_2_ under septic conditions (neutrophils/phagocytes, endothelial cells and plasma), RBCs are dependent on antioxidant scavenging mechanisms to survive oxidative stresses and remain viable. 

#### 4.6.1. Effect of Sepsis on Erythrocyte Catalase

The enzyme catalase (CAT) decomposes H_2_O_2_ to water and oxygen [100]. In RBC lysates of septic patients at the time of study enrollment, Karpetsa et al. [101] found that catalase activity was increased and oxidized protein (assessed by carbonyl level) was decreased in sepsis survivors. There was no difference in RBC glutathione levels. Additionally, Guerreiro et al. [102] reported that plasma SOD was increased in sepsis non-survivors, and a fall in plasma SOD was associated with better outcomes. However, there was no difference in plasma catalase or Thiobarbituric acid reactive substances (TBARS) level (a general marker of oxidative stress) between survivors and non-survivors. Consistent with findings in the RBC lysates [101], plasma carbonyl level (indicator of protein oxidation) was elevated in non-survivors, suggesting that increased protein oxidation had occurred in both the plasma and the RBC by the time of sepsis diagnosis. These results were in partial agreement with an earlier study [103] that found sepsis increased both plasma SOD and CAT and RBC SOD and CAT activities at the time of diagnosis. However, while both plasma SOD and CAT activity remained elevated in sepsis non-survivors, there was no difference in RBC SOD and CAT activity in non-survivors.

#### 4.6.2. Effect of Sepsis on Erythrocyte Peroxiredoxin II

A second RBC peroxidase is the thiol-dependent peroxiredoxin II [104,105,106] (Prx2 or PRDX2), the third most abundant protein in the RBC, of which a small fraction binds to the inner leaflet of the cell membrane [105]. The high concentration of Prx2 in the RBC suggests that it functions to detoxify low levels of H_2_O_2_ that are generated through autoxidation mechanisms [107]. However, in vitro experiments, which exposed human RBCs to activated neutrophils (by lipopolysaccharide (LPS) or *Staphylococcus aureus* (*S. aureus*)) [104], revealed that RBC Prx2 oxidation increased rapidly to a maximum by 30 min, before decreasing at 60 min. In these experiments, catalase (which detoxifies hydrogen peroxide) had a greater inhibitory effect on Prx2 oxidation than superoxide dismutase (which detoxifies superoxide anion. In whole animal experiments, RBC Prx2 oxidation in endotoxemic mice (injected with *E coli* LPS) increased rapidly from 6 h to a maximum at 10 h, after which Prx2 oxidation gradually decreased over the next 24 h. These results indicated that RBCs experienced considerable oxidative stress in response to activated neutrophils and endotoxemia.

**Figure 2:**
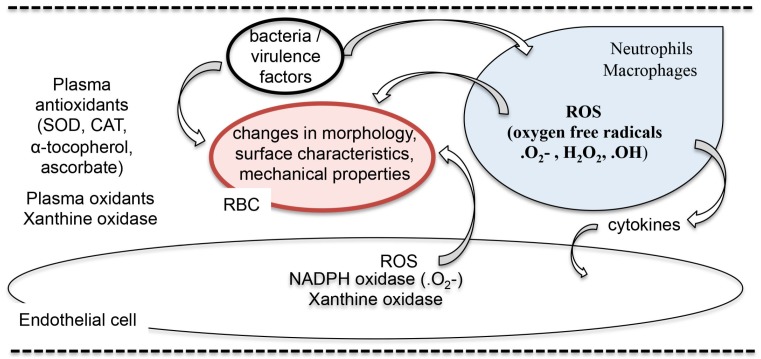
Interactions between erythrocytes, bacterial virulence factors, phagocytes and endothelial cells and sources of red blood cell (RBC) exposure to reactive oxygen species (ROS). The schematic depicts interactions between bacteria and virulence factors with erythrocytes and phagocytes. Phagocytes generate oxygen free radicals, release reactive oxygen species and activate endothelial cells (EC) that also release ROS, exposing RBCs to exogenous sources of ROS. The plasma contains oxidant generating enzymes and various antioxidants that act to scavenge ROS. In addition to exposure from exogenous ROS, the RBC is also exposed to endogenous sources of ROS via hemoglobin autoxidation. Abbreviations: SOD (superoxide dismutase), CAT (catalase), .O_2_^−^ (superoxide anion), H_2_O_2_ (hydrogen peroxide), .OH (hydroxyl radical).

Taken together, sepsis induces multiple sources of reactive oxygen species (neutrophils, endothelial cells, erythrocytes and plasma) and is associated with decreased antioxidant status. Several studies have linked leukocyte (neutrophil) ROS with altered erythrocyte properties and in vitro studies have offered insight into possible ROS mediated effects. However, more definitive sepsis studies are required to determine how the overall antioxidant system in the erythrocyte is altered during sepsis-induced oxidant stress and how antioxidant repletion affects both erythrocyte antioxidant mechanisms and erythrocyte function. See Figure 2 for a schematic of RBC exposure to exogenous sources of reactive oxygen species.

## 5. Sepsis Increases Intracellular Erythrocyte Ca^2+^

Intracellular calcium plays an important role in the RBC, where Ca^2+^ modulates membrane leaflet phosphatidylserine distribution (a signal associated with RBC clearance), proteolysis (calpain activation), transglutaminase activity, RBC morphology, cell volume, rheology and adhesion. Accordingly, Ca^2+^ concentration is tightly regulated in the RBC via a plasma membrane calcium ATPase pump (PMCA) that maintains a large Ca^2+^ gradient across the RBC membrane (1.8 mM in plasma vs. a 30–60 nm concentration in RBC cytoplasm) [108].

### Sepsis and Endotoxemia Alter Intracellular RBC Ca^2+^ Homeostasis

In a sepsis clinical study, Desai et al. [109] found a strong correlation between sepsis and hypocalcemia, with sepsis non-survivors having decreased plasma calcium compared to survivors. However, Todd et al. [110] reported that intracellular RBC Ca^2+^ levels were increased in sepsis patients. In the same study, these authors were able to reproduce the clinical findings in vitro by incubating human whole blood with *E. coli* endotoxin. The increased RBC intracellular Ca^2+^ was not inhibited by verapamil (Ca^2+^ channel blocker) but was inhibited by pretreatment with ATP, suggesting an energy deficiency was responsible for the increased Ca^2+^. In a separate study, Ruef et al. [111] incubated RBCs from healthy donors with *E. coli* Lipid A (innermost region of lipopolysaccharide endotoxin) and found it increased RBC cytosolic Ca^2+^ levels and decreased RBC deformability. Additionally, the effects of Lipid A on RBC rheology could be inhibited by verapamil and staurosporine (protein kinase inhibitor), but not by inhibiting rho-kinase. Furthermore, consistent with increased intracellular Ca^2+^, Lau et al. [112] reported that RBC Ca^2+^ pump activity (Vmax) decreased in septic patients. In vitro experiments [113] have associated decreased intracellular Ca^2+^ with altered RBC morphology (echinocyte formation) and decreased RBC deformability.

## 6. A Hallmark of Sepsis Is Increased Capillary Stopped-Flow

A hallmark of sepsis in both experimental models [14,15,114] and clinical patients [16,22] is a high degree of capillary stopped flow (Figure 1). While the in vivo mechanism of capillary stopped flow has not been completely elaborated, in vivo and in vitro evidence suggests that several factors may be involved: (1) RBC adhesion to endothelial cells, mediated by LPS and intermittent flow conditions [115], increased RBC intracellular Ca^2+^ and redistribution of phosphatidylserine (PS) to the outer RBC membrane leaflet [116], interaction with endothelial chemokine ligand [117] and matrix thrombospondin [118]; (2) decreased RBC deformability [3]; (3) increased platelet adhesion [18]; and (4) increased coagulation [28]. While sepsis induced decreased RBC deformability may be a factor in capillary stopped-flow [3], the septic spleen will likely trap the most rigid RBCs, as its smaller and shorter interendothelial slit dimensions [38,39] makes it more sensitive to RBCs with abnormal morphology and decreased deformability than skeletal muscle capillaries, which have a diameter of approximately 5–6 µm.

## 7. The Effect of Sepsis and Bacterial Virulence Factors on RBC Physiology and Survival

A variety of studies have shown that sepsis in general [119] and bacterial virulence factors in particular, including pyocyanin (*Pseudomonas aeruginosa*, *(P. aeruginosa*) [28]), peptidoglycan [120,121], lipopeptides [122,123], sphingomyelinase [124], α-haemolysin (*E. coli*) [125], and listeriolysin (*Listeria monocytogenes*) [126], can induce a redistribution of the RBC membrane phospholipid phosphatidylserine from the inner to the outer membrane leaflet, generating an external RBC signal associated with senescence [127], eryptosis and increased RBC clearance [128].

### 7.1. Pyocyanin Decreases RBC Survival

An example of the damaging effect on the RBC, and possible sequence of events caused by a virulence factor, is illustrated by pyocyanin. Pyocyanin (PCN) a redox-active metabolite released by *P. aeruginosa*. It exists in both oxidized and reduced states, and is associated with the production of reactive oxygen species. Inside cells, it mediates the reduction of oxygen to superoxide anion via the oxidation of NADPH to NADP^+^. Additionally, it inactivates catalase. Taken together, PCN induces the generation of oxygen free radicals, while inhibiting an important antioxidant enzyme, which can result in hydrogen peroxide oxidant stress. In the lung, the tissue responds by upregulating the enzyme antioxidants catalase and superoxide dismutase; however, the RBC lacks this ability. In isolated human RBCs treated with PCN, Qadri et al. [28] detected: (1) increased ROS levels (assessed by fluorescence);(2) evidence of increased cytosolic Ca^2+^ (increased percent of Ca^2+^ dye loaded cells and cleavage of Ca^2+^-dependent calpain); (3) RBC shrinkage (dehydration); (4) increased ceramide formation (membrane phospholipid); (5) increased exposure of phosphatidylserine on the outer RBC membrane leaflet; and (6) evidence of increased clearance of labeled PCN treated RBCs from the plasma and accumulation of PCN treated RBCs in the spleen. Additionally, when isolated human RBCs were incubated with plasma from septic patients positive for *P. aeruginosa*, the RBCs reproduced the in vitro PCN RBC phenotype with increased redistribution of PS from the inner to the outer membrane leaflet.

### 7.2. Bacterial Neuraminidase Is Elevated in Septic Patients and Decreases RBC Survival in Animal Models

The erythrocyte membrane contains glycoproteins that have sialic acid attached to their extracellular ends that give the RBC surface a negative charge (sialic acid is a monosaccharide derivative of neuraminic acid with a nine-carbon backbone). Bacterial neuraminidase enzymes, released by bacteria including *Pseudomonas aeruginosa*, *Clostridium perfringens* and *Vibrio cholera*, are glycoside hydrolases that cleave sialic residues and reduce membrane surface charge. Some bacteria are known to use sialic acid as a carbon and nitrogen source. 

In two clinical studies comparing critically ill ICU non-septic and septic patients (with decreased HCT, Hb and RBC count), Piagnerelli et al. reported both increased blood neuraminidase activity [7] and increased levels of plasma sialic acid [5] in septic patients. Both neuraminidase activity and sialic acid were elevated in critically ill non-septic patients compared to control and neuraminidase activity was found to correlate with C-reactive protein in all critically-ill patients. Morphologically, septic RBCs were found to be more spherical [5], consistent with the observation that neuraminidase treated RBCs slowly change from a discocytic to a stomatocytic shape [129], see Figure 1E. In vitro experiments, where isolated RBCs were incubated with *Clostridium perfingens* neuraminidase, demonstrated that RBC shape correlated with the extent of sialic cleavage [7]. Desialyation exposes underlying antigenic galactose residues that leads to RBC clearance [127].

In experimental studies where isolated RBCs from different species (human, rat and rabbit) were incubated with *Vibrio cholera* neuraminidase, Durocher et al. [130] found decreases in RBC sialic acid content and surface charge, but no change in RBC volume, deformability or ATP level. This neuraminidase RBC phenotype was similar in all species. Polyacrylaimde gel analysis of RBC membrane proteins revealed no change in membrane proteins, but significant changes in glycoproteins. Agglutination tests with soybean agglutinin revealed dose-dependent differences in agglutination with human and rat, but not rabbit neuraminidase treated RBCs. This loss of RBC surface charge may be a factor in sepsis induced RBC aggregation, see Figure 1D. When ^51^Cr-labeled neuraminidase treated RBCs were injected into rats and rabbits, they were cleared rapidly and found to accumulate predominantly in the liver.

## 8. Alterations in RBC Membrane Proteins in Critically Ill Patients and Trauma Animal Models

In a study of 24 critically-ill patients, 15 with sepsis or septic shock and nine without sepsis, and 10 healthy volunteers, Piagnerelli et al. [131] reported that at the time of admission, critically-ill patients had decreased RBC, HCT and Hb and increased MCV (mean corpuscular volume) compared to control, with no differences between sepsis and non-sepsis patients. CRP, however, was elevated in sepsis patients compared to non-septic critically ill patients and control. In addition, on Day 1, the amount of spectrin (cytoskeleton protein) was decreased in non-septic patient RBCs compared to control. No differences in Band 3 or protein 4.1 content were detected. Expressed as ratios, the authors reported increases in Band 3/spectrin and Protein 4.2/band 3, and decreases in spectrin, ankyrin, Band 3, Protein 4.1 and Protein 4.2 relative to actin in critically ill patients, but there were no differences between sepsis and non-sepsis critically ill patients. 

By comparison, in a 4.5 h animal model of trauma (T) and hemorrhagic shock (HS), where rats underwent a laparotomy followed by 90 hemorrhagic shock (T/HS, mean arterial pressure 30–35 mmHg) Caprio et al. [132] reported that T/HS compared to trauma without hemorrhagic shock, altered RBC morphology (echinocyte and spheroechinocyte formation) and rheology (decreased RBC deformability). While no change in RBC membrane protein (α,β-spectrin, ankyrin, band 3, protein 4.1/4.2, protein 4.9 and actin) content was detected, serine phosphorylation increased in β-spectrin and ubiquitination decreased in α-spectrin.

### Sepsis Induces Band 3 Phosphorylation 

In an experimental study to assess the effect of sepsis on RBC band 3 phosphorylation, Condon et al. [133] conducted both in vitro and in vivo experiments on mouse RBCs. In preliminary experiments, they treated isolated mouse RBCs with pervanadate, a phosphotyrosine phosphatase (PTP) inhibitor, for 10 min at 4 °C. The resulting (and expected) increase in band 3 tyrosine phosphorylation was associated with decreased RBC deformability and impaired sulfate uptake, a marker of anion exchange function. Next, they used an in vivo mouse model of sepsis with a 70% mortality at 72–96 h, to assess the effect of polymicrobial sepsis on RBC band 3 tyrosine phosphorylation at 24 h. The authors found no change in isolated RBC band 3 content between sepsis and control, but did find increased normalized band 3 tyrosine phosphorylation in the sepsis mice. There was no difference in serine phosphorylation between sepsis and control. Additionally, in contrast to the in vitro study, they did not detect any change in either anion exchange activity or in band 3 inhibition assays. A fluorescence assay suggested that sepsis induced an organizational or structural change in band 3 (possibly related to band 3 clustering).

## 9. Sepsis Impairs RBC O_2_-Dependent ATP Efflux

The microcirculation is a highly integrated functional system [134] that distributes RBCs throughout skeletal muscle and other tissues via capillary networks. Blood flow into the capillaries is controlled by the upstream arteriolar tone feeding the capillary network. Vasoactive molecules released from the RBC in response to hypoxic conditions, including ATP [135,136], nitric oxide (generated by deoxy-hemoglobin acting as a nitrite reducatse) [137,138,139] and nitrosothiols [140] have been proposed as possible mediators of hypoxic vasodilation; though, the role of nitric oxide vis-à-vis nitrosothiols and reaction with the hemoglobin βCys93 residue is somewhat controversial [141,142,143]. As RBCs travel through the microcirculation, ATP may be released in hypoxic regions from ATP compartments [144] via the RBC Pannexin-1 channel [145]. ATP then diffuses to the vascular endothelial cells where it binds to P2Y receptors and triggers a conducted vascular response back through the capillaries to the arterioles, resulting in local vasodilation [136].

**Figure 3:**
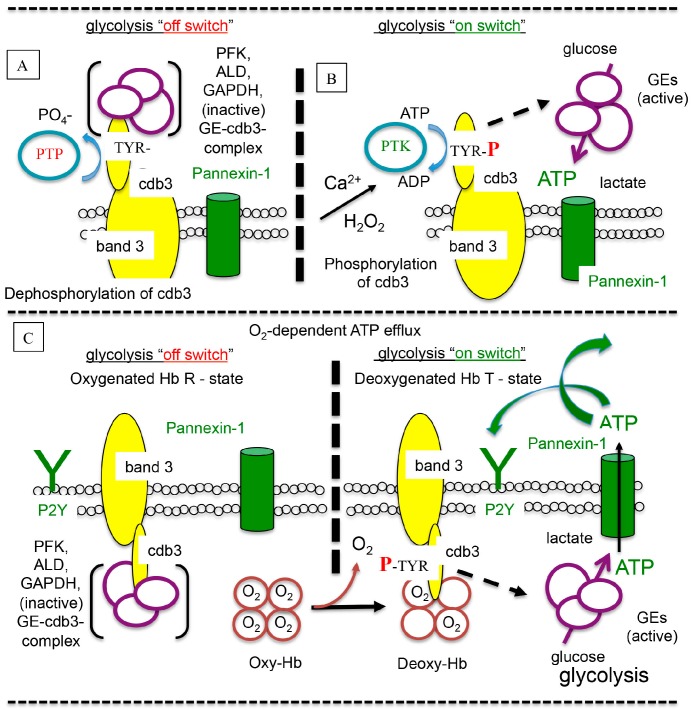
Glycolysis is regulated at the RBC membrane via molecular switch (on/off) mechanisms involving phosphorylation, deoxyhemoglobin (Deoxy-Hb) and a complex of inactive glycolytic enzymes (GEs) [151,153] bound to the cytoplasmic domain of band 3 (cdb3). (**A**) phosphotyrosine phosphatase (PTP) maintains cdb3 in a dephosphorylated state [154]. (**B**) Hydrogen peroxide (H_2_O_2_) [155] and Ca^2+^ induce band 3 phosphorylation by disassociating PTP from band 3 [156] allowing a phosphotyrosine kinase (PTK) to then phosphorylate band 3. Tyrosine phosphorylation of band 3 leads to the displacement of GEs (broken arrow) and increased glycolysis [155]. (**C**) Similarly, deoxygenation of oxyhemoglobin (oxy-Hb) to deoxy-Hb (solid arrow) is associated with band 3 phosphorylation [157]. Moreover, deoxy-hemoglobin binding to cdb3 [158] is associated with release of GEs (broken arrow), glycolysis and increased release of adenosine triphosphate (ATP) [135]. Sepsis impairs this mechanism and RBC O_2_-dependent ATP efflux [13]. Abbreviations: PFK (phosphofructokinase), ALD (aldolase), GAPDH (glyceraldehyde 3-phosphate dehydrogenase), ADP (adenosine diphosphate), TYR (tyrosine), TYR-P (tyrosine-phosphate). (Modified from Bateman et al. [13], available online: https://creativecommons.org/licenses/by/4.0/).

In vitro evidence using isolated human [146] and rat RBCs suggests that O_2_-dependent ATP release is mediated by deoxyhemoglobin and glycolysis [135], consistent with the hypothesis that deoxyhemoglobin displaces glycolytic enzymes from the cytoplasmic domain of band 3 (cdb3) [147,148,149,150,151] resulting in a local increase in glycolysis at the inner membrane and release of ATP from the cell, see Figure 3. Moreover, in whole blood from a 6 h rat model of hypotensive sepsis, Bateman et al. [13] found that RBC O_2_-dependent ATP efflux was impaired. This loss of RBC function was associated with reduced plasma pH, increased plasma lactate and nitric oxide (measured as nitrite + nitrate (NO_2_^−^ + NO_3_^−^), increased capillary stopped flow and a delayed capillary response to hypoxic capillaries. While the mechanism is uncertain, Rozier et al. [152] have demonstrated in isolated rabbit RBCs that increased lactate inhibits RBC O_2_-dependent ATP release.

## 10. Conclusions

Sepsis induces a wide range of effects on the erythrocyte. Clinical and experimental studies have shown that sepsis alters the heterogeneity of red cell distribution width (RDW), although its reported association with mortality has been questioned [45,49]. Older studies reported that sepsis could left-shift the hemoglobin oxygen dissociation curve, but that this was preventable by avoiding hypophosphatemia, transfusion with 2,3-BPG depleted blood and treating acidemia [62]. Furthermore, sepsis-induced alterations in erythrocyte morphology and especially rheology (RBC deformability) have been reported to be early indicators of sepsis [3,32]. Intriguingly, a few reports have suggested that a small subpopulation of older erythrocytes may be at greater risk of decreased deformability [3,40]. Sepsis-induced oxidant stress arises from multiple sources including activated neutrophils, endothelial cells, plasma and potentially, under certain conditions the erythrocyte itself via an autoxidation mechanism [18,24,85,93]. The oxygen free radicals superoxide anion and hydroxyl radical and the reactive oxygen species hydrogen peroxide can all induce deleterious reactions affecting RBC proteins, cytoskeleton and the RBC membrane, all of which can impact RBC function [95,96,97]. Additionally, bacterial virulence factors utilize different mechanisms that result in rapid deterioration and clearance of affected erythrocytes [7,28]. Furthermore, while sepsis impairs the erythrocyte O_2_-dependent ATP signaling response to hypoxia, the mechanism is unknown [13].

Evidence from sepsis studies (and controlled in vitro studies), especially those that investigate specific virulence factors help to isolate and establish a sequence of events or mechanisms that alter erythrocyte biochemistry and ultimately lead to altered erythrocyte morphology, rheology and premature destruction. While evidence suggests that oxidant stress leads to increased intracellular calcium [110] that triggers a host of calcium mediated deleterious effects, the precise pathological mechanism involved is unknown; though, it may involve the plasma membrane calcium ATPase pump [112]. 

The oxidant stresses that impinge on the septic erythrocyte are a significant cause of RBC damage and deterioration. The reactions are complex and vary depending on the severity of sepsis and the location of the erythrocyte, whether it is free flowing in the systemic circulation, adjacent to oxidatively active neutrophils or endothelial cells or even trapped in stopped-flow capillaries in the septic microcirculation. Important issues to be addressed regarding the septic erythrocyte include understanding the mechanisms of virulence factors and whether sepsis-induced injury results from strictly exogenous reactive oxygen species or does RBC autoxidation play an increasing role in the septic RBC as RBCs become trapped in the septic microcirculation or experience increased exposure to local regions of tissue hypoxia [21,23].

Clinical evidence presented in this review indicted that some antioxidants were altered to a greater extent in plasma than the RBC [103], suggesting that plasma antioxidant repletion or augmentation might have beneficial effects on overall antioxidant status, which numerous reports have shown is decreased or even deficient in sepsis patients. Indeed, reports that the small molecule antioxidants, α-tocopherol and ascorbic acid [73,75], can protect erythrocyte deformability and improve septic patient outcome support increased research in this area.

## Abbreviations

2,3-BPG/2,3-DPG	2,3-bisphosphoglycerate
ATP	Adenosine triphosphate
CAT	Catalase
H_2_O_2_	Hydrogen peroxide
MDA	Malonyldialdehyde
NADH	Nicotinamide adenine dinucleotide
NADPH	Reduced nicotinamide adenine dinucleotide phosphate
PS	Phosphatidylserine
PTP	Phosphotyrosine phosphatase
PIK	Phosphotyrosine kinase
ROS	Reactive oxygen species
O_2_^−^	Superoxide anion
SOD	Superoxide dismutase
TBARS	Thiobarbituric acid reactive substances
